# Xanol Promotes Apoptosis and Autophagy and Inhibits Necroptosis and Metastasis via the Inhibition of AKT Signaling in Human Oral Squamous Cell Carcinoma

**DOI:** 10.3390/cells12131768

**Published:** 2023-07-03

**Authors:** Hyung-Mun Yun, Bomi Kim, Soo Hyun Kim, Seung-Hae Kwon, Kyung-Ran Park

**Affiliations:** 1 Department of Oral and Maxillofacial Pathology, School of Dentistry, Kyung Hee University, Seoul 02447, Republic of Korea; yunhm@khu.ac.kr; 2 National Development Institute for Korean Medicine, Gyeongsan 38540, Republic of Korea; bom0203@nikom.or.kr (B.K.); beluga81@nikom.or.kr (S.H.K.); 3 Korea Basic Science Institute (KBSI), Seoul 02841, Republic of Korea; kwonsh@kbsi.re.kr; 4 Korea Basic Science Institute (KBSI), Gwangju 61751, Republic of Korea

**Keywords:** *A. keiskei*, apoptosis, autophagy, necroptosis, OSCC, xanol

## Abstract

*Angelica keiskei* Koidzumi (*A. keiskei*) is used as a traditional medicine, anti-aging agent, and health food, as well as to restore vitality. Xanthoangelol (xanol), a prenylated chalcone, is the predominant constituent of *A. keiskei*. Oral squamous cell carcinoma (OSCC), the most common malignancy, has a high proliferation rate and frequent metastasis. However, it is unknown whether xanol has anti-OSCC effects on apoptosis, autophagy, and necroptosis. In the present study, we purified xanol from *A. keiskei* and demonstrated that it suppressed cell proliferation and induced cytotoxicity in human OSCC. Xanol triggered apoptotic cell death by regulating apoptotic machinery molecules but inhibited necroptotic cell death by dephosphorylating the necroptotic machinery molecules RIP1, RIP3, and MLKL in human OSCC. We also found that xanol inhibited the PI3K/AKT/mTOR/p70S6K pathway and induced autophagosome formation by enhancing beclin-1 and LC3 expression levels and reducing p62 expression levels. Furthermore, we showed that xanol prevented the metastatic phenotypes of human OSCC by inhibiting migration and invasion via the reduction of MMP13 and VEGF. Finally, we demonstrated that xanol exerted anticancer effects on tumorigenicity associated with its transformed properties. Taken together, these findings demonstrate the anticancer effects and biological mechanism of action of xanol as an effective phytomedicine for human OSCC.

## 1. Introduction

Oral squamous cell carcinoma (OSCC) is the most common malignancy, with a global incidence of approximately 377,000 and a mortality rate of 177,000 [1]. In the early stages, the 5-year survival rate is greater than 80%, whereas in the late stages, it decreases to less than 30%. More than 60% of OSCC cases have high morbidity and death rates as they are detected at a late stage [1,2]. Traditional OSCC therapies, including surgery, radiotherapy, and chemotherapy, have advanced but have not improved survival due to the aggressive characteristics of OSCC, including growth, invasion, metastasis, and recurrence [3,4]. Therefore, research into cell biology and pathophysiology that control the aggressive characteristics of OSCC will provide more knowledge for the development of innovative therapeutics.

*Angelica keiskei* Koidzumi (*A. keiskei*), which belongs to the family Umbelliferae, is a hardy perennial that grows mainly in Asian countries, including Korea, China, and Japan [5]. It is known as Shin-Sun Cho, which translates to “a precious herb used by God”, and is also known as tomorrow’s leaf because of its quick growth and capacity for regeneration [6]. For hundreds of years, *A. keiskei* has been used medicinally as an anti-aging agent and general health enhancer and as a daily health food in vegetable juice, tea, flour, wine, and cosmetics [6]. Recent research has confirmed many of the traditional uses against various diseases, such as Alzheimer’s disease, blood disorders, cancer, coronary heart disease, diabetes, gastrointestinal diseases, hypertension, and ischemia [6,7,8,9,10,11]. Therefore, *A. keiskei* is an important resource for the development of innovative drugs.

Xanthoangelol (xanol) is the major prenylated chalcone isolated from *A. keiskei*. Prenylated chalcones have drawn increased attention in the fields of nutrition and cancer prevention because of their pharmacological effects, which have been thoroughly studied in vitro and in preclinical investigations [12]. Xanol is known to have protective effects against allergic diabetes, inflammation, infection, ischemia, neurodegeneration, muscle loss, oxidative stress, tumor growth, and tumor metastasis [6,7,13,14]. However, the pharmacological and molecular effects of xanol on OSCC have not yet been investigated. In the present study, we purified xanol (>99.9% purity) from *A. keiskei*, studied its pharmacological and molecular activities, and discovered that it has anticancer effects against YD-10B cells, which were established from tongue cancer tissues of patients with OSCC.

## 2. Materials and Methods

### 2.1. Plant Material and General Experimental Procedures

*A. keiskei* was purchased at a commercial herbal medicine market. A voucher specimen (P598) was deposited in the Natural Products Bank of the National Institute for Korean Medicine Development. Organic solvents, including methanol (MeOH), *n*-hexane (Hx), ethyl acetate (EtOAc), and chloroform (CHCl_3_), were purchased from Duksan Chemical Co. (Seoul, Republic of Korea). Column chromatography was performed using silica gel 60 (70–230 mesh ASTM; Merck, Darmstadt, Germany), ODS-A (12 nm, S-75 m; YMC, Tokyo, Japan), and Sephadex LH-20 gel (GE Healthcare, Uppsala, Sweden). Preparative thin-layer chromatography was performed using 20 × 20 cm plates coated with 1 mm thick F254 silica gel (Merck). Nuclear magnetic resonance (NMR) spectra were recorded on a JEOL ECX-500 spectrometer operating at 500 MHz for ^1^H NMR and 125 MHz for ^13^C NMR (JEOL Ltd., Tokyo, Japan). High-performance liquid chromatography (HPLC) spectra were recorded on an Agilent 1260 series system (Agilent Inc., Palo Alto, CA, USA) with a photodiode array and an evaporative light-scattering detector.

### 2.2. Xanol Compound and Stock Solution

Xanol had the following characteristics: yellow powder; molecular formula, C_25_H_28_O_4_; ^1^H-NMR (500 MHz, CDCl_3_) δ 7.81 (1H, d, J = 15.2 Hz, H-β), 7.70 (1H, d, J = 9.0 Hz, H-6′), 7.52 (2H, d, J = 8.6 Hz, H-2, H-6), 7.44 (1H, d, =15.2 Hz, H-α), 6.86 (2H, d, J = 8.6 Hz, H-3, H-5), 6.41 (1H, d, J = 8.9 Hz, H-5′), 5.28 (1H, m, H-2″), 5.04 (1H, m, H-6″), 3.47 (2H, d, J = 7.0 Hz, H-1″), 2.08 (4H, m, H-5″, H-4″), 1.81 (3H, s, H-10″), 1.66 (3H, s, H-8″), 1.57 (3H, s, H-9″); and ^13^C-NMR (125 MHz, CDCl_3_) δ 192.5 (C=O), 164.0 (C-4′), 162.1 (C-2′), 158.3 (C-4), 144.4 (C-β), 140.0 (C-3″), 132.3 (C-7″), 130.8 (C-2, C-6), 129.5 (C-6′), 127.9 (C-1), 123.9 (C-6″), 121.1 (C-2″), 118.2 (C-α), 116.3 (C-3) 116.2 (C-5), 114.3 (C-1′), 114.2 (C-3′), 108.2 (C-5′), 39.9 (C-4″), 26.5 (C-5″), 25.9 (C-8″), 21.9 (C-1″), 17.9 (C-9″), 16.5 (C-10″). Xanol stock solution was dissolved in 100% dimethyl sulfoxide (DMSO). The DMSO concentration in the final xanol concentration was 0.1%.

### 2.3. Cell Culture

Human OSCC cells (YD-10B) were obtained from the Korean Cell Line Bank (Seoul, Republic of Korea). The cells were cultured in Dulbecco’s modified Eagle medium supplemented with heat-inactivated 10% fetal bovine serum and 1X Gibco^®^ Antibiotic-Antimycotic (Thermo Fisher Scientific, Waltham, MA, USA), and were incubated in a humidified atmosphere of 37 °C and 5% CO_2_.

### 2.4. 3-[4,5-dimethylthiazol-2-yl]-2,5-diphenyltetrazolium Bromide (MTT) Assay

Cells were seeded in 96-well plates for 24 h and incubated with xanol for 24 h. MTT assays were performed as previously described [15].

### 2.5. Western Blotting Analysis

Western blotting analysis was performed as previously described [16]. Antibodies against the following proteins were used: Cleaved-caspase-3 (1:1000, #9661), PARP (1:1000, #9542), Bcl-2 (1:1000, #15071), survivin (1:1000, #2808), p-MLKL (1:1000, #91689), MLKL (1:1000, #14993), p-RIP3 (1:1000, #93654,) RIP3 (1:1000, #13526), p-RIP1 (1:1000, #65746), RIP1 (1:1000, #3493), LC3A/B (1:1000, #12741), beclin1 (1:1000, #3495), p62 (1:1000, #5114), p-PI3K (1:1000, #4228), PI3K (1:1000, #4257), p-AKT (1:1000, #4060), AKT (1:1000, #4691), p-mTOR (1:1000, #2974), mTOR (1:1000, #2983), p-p70S6K (1:1000, #9204), and p70S6K (1:1000, #2708), all from Cell Signaling Technology (Beverly, MA, USA); Cyclin D1 (1:1000, #sc-20044), β-actin (C4, 1:1000, #sc-47778), Cdk4 (1:1000, #sc-23896), and Cdk6 (1:1000, #sc-7961), all from Santa Cruz Biotechnology (Santa Cruz, CA, USA); and MMP13 (1:1000, NBP1-45723) from Novus Biologicals (Centennial, CO, USA). Protein bands were detected using the ProteinSimple detection system (ProteinSimple Inc., Santa Clara, CA, USA).

### 2.6. Apoptosis Assay

Annexin V-stained apoptotic cells were detected using Annexin V-FITC Apoptosis Detection Kit (Biovision, Waltham, MA, USA), and apoptotic DNA fragmentation was analyzed using the in situ Cell Death Detection Kit (Roche Diagnostics GmbH, Mannheim, Germany) as described previously [15]. Images were observed under a fluorescence microscope and confocal microscope (STELLARIS 8, Leica Microsystems, Wetzlar, Germany) at the KBSI Seoul Center.

### 2.7. Immunofluorescence Assay

Immunofluorescence assays were performed as previously described [17]. Immunofluorescence was observed using,16 an IX73 inverted microscope (Olympus Corporation, Tokyo, Japan).

### 2.8. Autophagosome Detection Assay

An autophagosome detection assay was performed using the DAPGreen Autophagy Detection Kit (Dojindo, Kumamoto, Japan) as previously described [15]. Autophagosome formation was observed under an Olympus IX73 inverted microscope (Olympus Corporation).

### 2.9. Cell Migration and Invasion Assays

Cell migration and invasion assays were performed using wound healing and Boyden chamber experiments, respectively, as previously described [15]. Cell migration to the wounded region and invasion across the Matrigel-coated membrane were observed using an AE2000 light microscope (Motic, Schertz, TX, USA).

### 2.10. Soft Agar Colony Formation Assay

The soft agar colony formation assay was performed as previously described [17]. Anchorage-independent colonies were observed and quantified using an AE2000 light microscope (Motic).

### 2.11. Statistical Analysis

All numerical values were analyzed using GraphPad Prism version 5 (GraphPad Software, Inc., San Diego, CA, USA). Statistical significance (*p* < 0.05) was analyzed using unpaired Student’s *t*-test and data in graphs are shown as the mean ± standard error of the mean.

## 3. Results

### 3.1. Xanol Isolated from MeOH Extract of Dried A. keiskei Inhibits Cell Proliferation in Human OSCC

**Figure 1:**
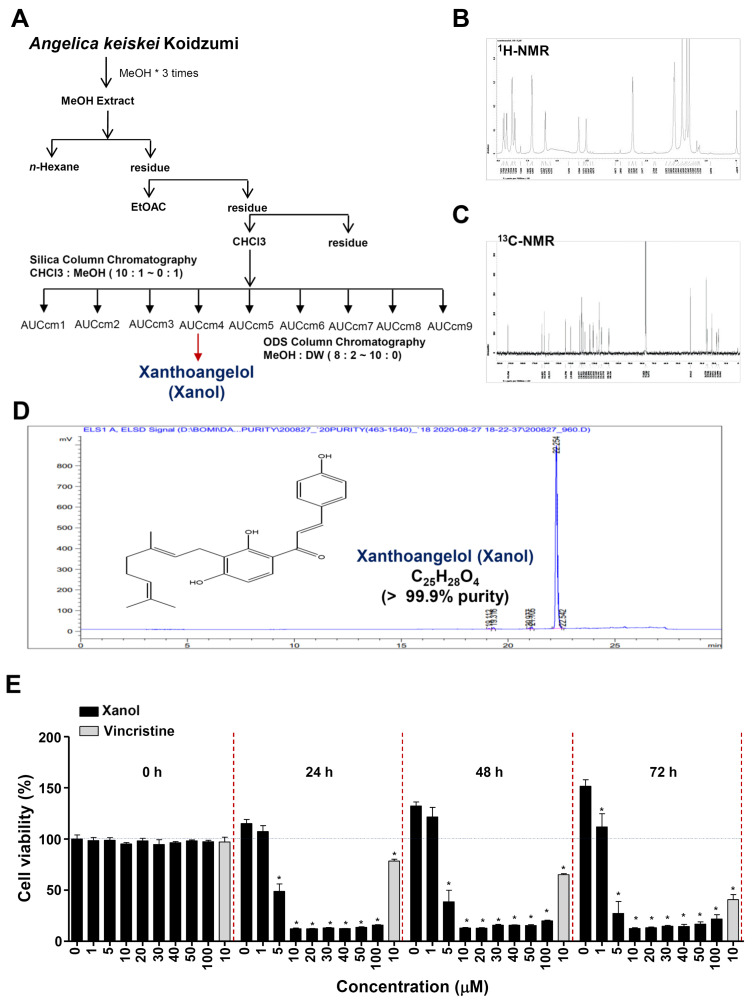
Purification of xanol from a MeOH extract of *Angelica keiskei* Koidzumi and its effects on cell proliferation. (**A**) Isolation scheme of xanol purified from *A. keiskei*. (**B**,**C**) The ^1^H-nuclear magnetic resonance (NMR; 500 MHz, CDCl_3_) spectrum (**B**) and ^13^C-NMR (125 MHz, CDCl_3_) spectrum (**C**) of xanol. (**D**) High-performance liquid chromatography results and structure of xanol. (**E**) Human oral squamous cell carcinoma (OSCC) cells were treated for 0–72 h with 1–100 μM concentrations of xanol, and cell proliferation was analyzed using MTT assay. Data are representative of three independent experiments. Statistical significance was set at *p* < 0.05 * and data were analyzed using GraphPad Prism version 5.

Dried *A. keiskei* (4700 g) was extracted using MeOH at room temperature over 2 days. The MeOH extract (1671.65 g) was evaporated to dryness and suspended in 1700 mL of distilled water (DW), and the solvent was partitioned three times using Hx, CHCl_3,_ and EtOAc. The CHCl_3_-soluble fraction (28.61 g) was separated into nine fractions (AUCcm, 1–9) using chromatography on a silica gel column and eluted with a gradient of CHCl_3_ and MeOH (from 10:1 to 0:1). The fourth fraction was subjected to an ODS-A column and eluted with a gradient of MeOH and DW (80–100% MeOH). Xanol (110 mg) was obtained from the second fraction (Figure 1A). Figure 1B,C show the ^13^C-NMR (125 MHz, CDCl_3_) and ^1^H-NMR (500 MHz, CDCl_3_) spectra of xanol. The HPLC chromatogram and structure of xanol (molecular formula C_25_H_28_O_4_; purity >99.9%) are shown in Figure 1D. The structure of xanol was confirmed by comparing its spectral data with data from the literature [18]. To determine the anti-OSCC effects of xanol against human OSCC cell proliferation, various concentrations (0, 1, 5, 10, 20, 30, 40, 50, and 100 μM) of xanol were used to treat human OSCC cells. MTT assays were performed at 0, 24, 48, and 72 h. The results revealed that xanol significantly inhibited cell proliferation and induced cytotoxicity in human OSCC, and that xanol had a more effective inhibitory effect on OSCC than 10 μM vincristine (Figure 1E). Vincristine was used as a positive control because it is widely used as an anticancer agent. For subsequent analyses, human OSCC cells were treated with xanol concentrations of 1, 5, and 10 μM.

### 3.2. Xanol Triggers Apoptotic Cell Death and Inhibits Necroptotic Cell Death in Human OSCC

**Figure 2:**
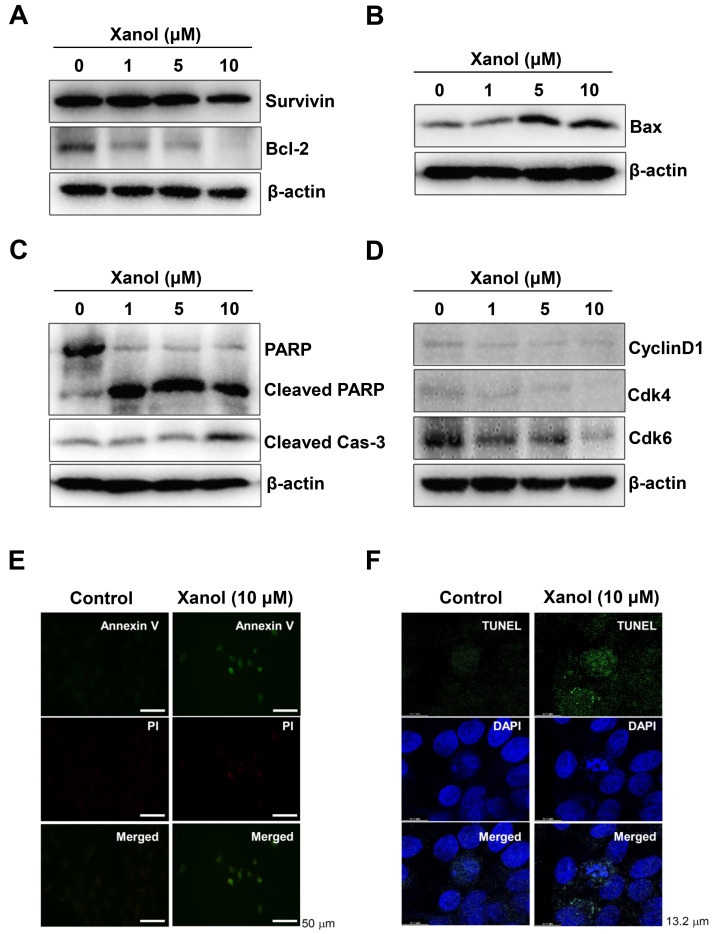

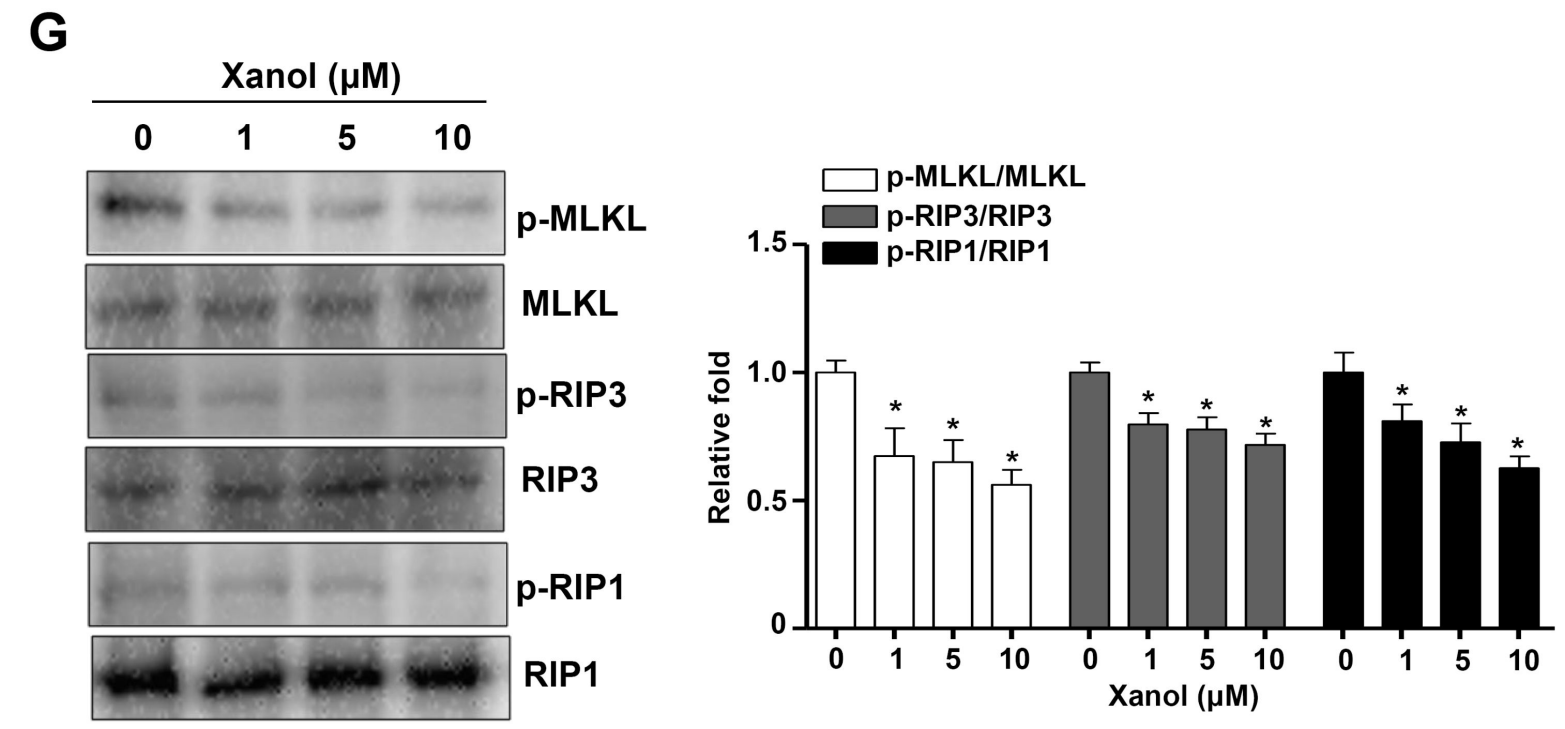
Effects of xanol on apoptosis and necroptosis in human OSCC. (**A**–**D**) Human OSCC cells were treated with xanol for 24 h at 1–10 μM concentrations, and the levels of apoptosis-related proteins were analyzed by western blotting with antibodies against surviving and Bcl-2 (**A**), Bax (**B**), PARP, cleaved PARP, cleaved caspase-3 (**C**), and cyclin D1, Cdk4, and Cdk6 (**D**). β-actin levels were determined as a loading control. (**E**,**F**) Human OSCC cells were treated with 10 μM xanol for 24 h, and the Annexin V (green color) and Propidium Iodide (PI, red color) were observed using an immunofluorescence microscope (**E**). TUNEL (green color) and 4′,6-Diamidino-2-phenylindole (DAPI, blue color) were observed using a confocal microscope (**F**). (**G**) Human OSCC cells were treated with xanol for 24 h at 1–10 μM concentrations, and necroptosis-regulatory proteins were analyzed by western blotting with antibodies against p-MLKL (Ser358), MLKL, p-RIP3 (Ser227), RIP3, p-RIP1 (Ser166), and RIP1. Data are representative of three independent experiments. Statistical significance was set at *p* < 0.05 * and data were analyzed using GraphPad Prism version 5.

To explore whether apoptosis was involved in the anticancer activities induced by xanol, apoptotic machinery molecules were analyzed at the protein level in human OSCC cells after treatment with xanol for 24 h. Xanol decreased the levels of the pro-apoptotic proteins survivin and Bcl-2, whereas it elevated the levels of the anti-apoptotic protein Bax (Figure 2A,B). We then analyzed the cleavage of PARP and caspase-3, which are the main features of apoptosis, and the data revealed that xanol induced PARP and caspase-3 cleavage (Figure 2C). In addition, xanol decreased the levels of Cdk6, Cdk4, and Cyclin D1, which regulate cell proliferation by forming complexes, in human OSCC cells (Figure 2D). Xanol-mediated apoptotic cell death was validated using two apoptosis assays: Annexin V-FITC (plasma membrane change as an early apoptotic event) and TUNEL (DNA fragmentation as a late apoptotic event). As shown in Figure 2E,F, Annexin V- and TUNEL-positive cells were detected in human OSCC cells using xanol.

Next, we examined whether xanol affected the necroptotic molecular machinery in human OSCC. The data revealed that xanol inhibited the phosphorylation of mixed lineage kinase-domain-like pseudokinase (MLKL), receptor-interacting serine/threonine-protein kinase (RIP)1, and RIP3 (Figure 2G). These results suggest that xanol exerts anticancer effects on human OSCC by inducing apoptosis and suppressing necroptosis.

### 3.3. Xanol Inhibits the PI3K-AKT-mTOR-p70S6K Pathway and Induces Autophagy in Human OSCC

**Figure 3:**
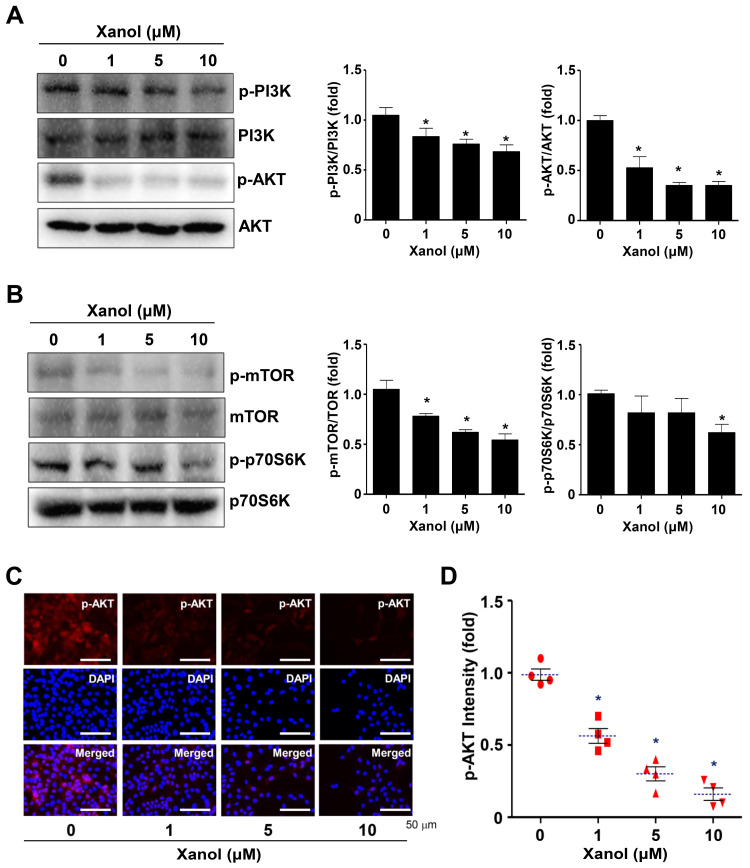
Effects of xanol on the AKT signaling pathway in human OSCC cells. (**A**,**B**) Human OSCC cells were treated with xanol for 24 h at 1–10 μM concentrations, and p-PI3K (Tyr458), PI3K, p-AKT (Ser473), and AKT (**A**) and p-mTOR (Ser2481), mTOR, p-p70S6K (Thr421/Ser424), and p70S6K (**B**) were analyzed by western blotting. (**C**,**D**) p-AKT (red color) levels were observed using an immunofluorescence assay. 4′,6-Diamidino-2-phenylindole (DAPI, blue color) was used to detect the nuclei of human OSCC cells (**C**). pAKT intensity is shown as a bar graph. Red symbol: n number. Dotted line: mean value (**D**). Data are representative of three independent experiments. Statistical significance was set at *p* < 0.05 * and data were analyzed using GraphPad Prism version 5.

To determine the biological mechanism of the apoptotic cell death induced by xanol, AKT signaling was analyzed in human OSCC cells, because this pathway is a key target in therapeutic approaches and is frequently amplified in patients with OSCC [19,20]. The results revealed that xanol significantly decreased PI3K and AKT phosphorylation levels compared to the total protein levels in human OSCC cells (Figure 3A). Next, we investigated the downstream proteins and the results revealed that xanol significantly suppressed mTOR and p70S6K phosphorylation compared with the total protein levels in human OSCC cells (Figure 3B). This was further validated using fluorescence microscopy, which showed that xanol decreased AKT phosphorylation levels in human OSCC cells (Figure 3C,D).

**Figure 4:**
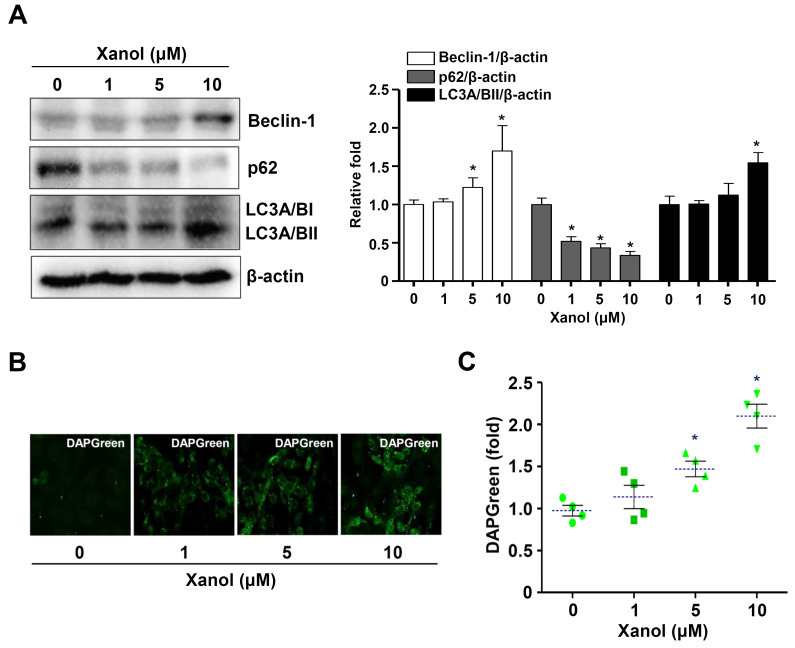
Effects of xanol on the autophagic process and autophagosome formation in human OSCC cells. (**A**) Human OSCC cells were treated with xanol for 24 h at 1–10 μM concentrations, and beclin1, p62, LC3A/BI, and LC3A/BII levels were analyzed by western blotting. (**B**,**C**) Autophagosome formation was analyzed using a DAPGreen Autophagy Detection kit and DAPGreen-positive autophagosomes were observed using a fluorescence microscope (**B**). DAPGreen intensity is shown as a bar graph. Green symbol: n number. Dotted line: mean value (**C**). Data are representative of three independent experiments. Statistical significance was set at *p* < 0.05 * and data were analyzed using GraphPad Prism version 5.

We next examined whether autophagy was involved in the xanol-mediated effects because the AKT pathway controls the autophagic molecular machinery and autophagosome formation. Western blotting analysis showed that xanol decreased p62 levels but increased microtubule-associated protein light chain 3A/B (LC3A/B) and beclin-1 protein levels (Figure 4A). Autophagosome formation was monitored using fluorescence microscopy and it was confirmed that xanol induced autophagy in human OSCC cells, indicating that it promoted apoptosis and induced cell death (Figure 4B,C). These results suggest that xanol exerts anticancer effects on human OSCC cells by inducing apoptosis and autophagy via the PI3K-AKT-mTOR-p70S6K pathway.

### 3.4. Xanol Suppresses the Migration, Invasion, and Tumorigenicity of Human OSCC

**Figure 5:**
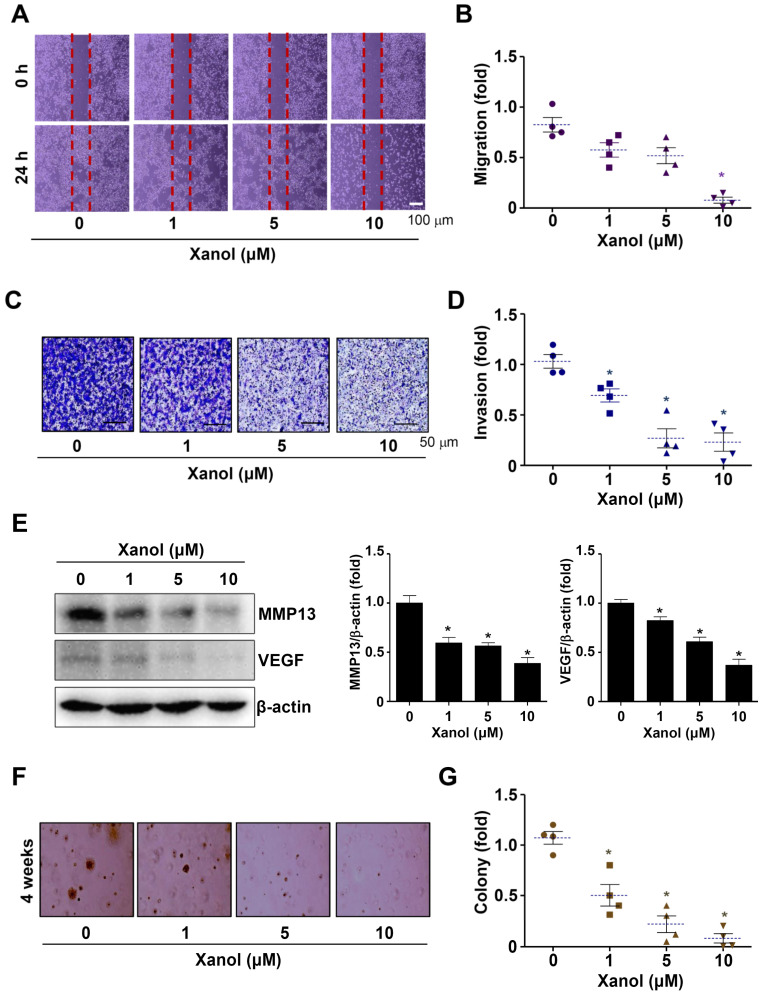
Effects of xanol on cell migration, invasion, and colony formation in human YD-10B OSCC cells. (**A**,**B**) Human OSCC cells were treated with 1–10 μM xanol concentrations for the indicated times, and cell migration was observed under a light microscope (**A**). The migration rate is shown as a bar graph (**B**). (**C**,**D**) Cell invasion was monitored using Boyden chamber assays, and invasive cells were detected under a light microscope (**C**). (**E**) MMP13 and VEGF levels were analyzed by western blotting. (**F**,**G**) Human OSCC cells were treated with 1–10 μM xanol concentrations for the indicated time, and colony formation was observed using a light microscope (**F**). Colony formation is shown as a bar graph (**G**). Symbol: n number. Dotted line: mean value. Data are representative of three independent experiments. Statistical significance was set at *p* < 0.05 * and data were analyzed using GraphPad Prism version 5.

Based on the molecular effects of xanol on human OSCC cells, we explored whether xanol exhibits anti-metastatic and anti-tumorigenic activities in human OSCC. Initially, a wound-healing experiment was performed to examine the migration of human OSCC cells. The results revealed that xanol substantially inhibited cell migration compared with the control (Figure 5A,B). Next, a Boyden chamber experiment was performed to examine the invasive ability of human OSCC cells via extracellular matrix degradation. The results revealed that xanol substantially inhibited membrane penetration compared to the control (Figure 5C,D). In addition, xanol decreased the levels of MMP13 and VEGF proteins, which are important regulatory proteins in metastasis, angiogenesis, and tumorigenesis (Figure 5E). Finally, soft agar colony formation was observed to determine tumorigenicity and transformed properties, and we found that xanol substantially suppressed the anchorage-independent growth of human OSCC cells compared to the control (Figure 5F,G). Our results suggest that xanol exerts anticancer effects on the metastasis and tumorigenicity of human OSCC cells.

## 4. Discussion

Approximately 50% of patients with OSCC survive; however, traditional OSCC therapies do not improve survival and have been linked to serious side effects [4,21]. OSCC is highly lethal and has high mortality rates because of its proliferation, invasion, metastasis, and chemotherapeutic resistance [22,23]. Thus, we purified the bioactive prenylated chalcone xanol to >99.9% purity from *A. keiskei* and demonstrated its anticancer effects against human OSCC.

Malignant tumors develop when cells with mutations in various oncogenes or tumor-suppressor genes evade cell death [24]. Various anticancer medications prevent cell proliferation by inducing apoptosis, which is a key biological mechanism underlying the anticancer effect. Here, we found that xanol prevented the proliferation of human OSCC cells and induced apoptotic cell death. Apoptotic cell death is caused by caspase-dependent cascades and is regulated by the Bcl-2 family (pro-apoptotic Bax and anti-apoptotic Bcl-2 proteins) and an inhibitor of apoptosis (survivin) [25,26,27]. We demonstrated that xanol promoted PARP and caspase-3 cleavage—hallmarks of apoptosis—while reducing Bcl-2 and survivin levels and increasing Bax levels in human OSCC. In addition, xanol inhibited the levels of proteins that control the cell cycle, including cyclin D1, Cdk4, and Cdk6. Our data suggested that xanol inhibited the growth and caused the death of human OSCC cells by inducing apoptosis.

Although apoptosis is the most well-known mechanism of cell death, necroptosis is a recently discovered mechanism of programmed cell death that occurs via a caspase-independent mechanism. Necroptosis is regarded as a “fail-safe” cell death mechanism that takes place when cells are unable to cause apoptosis or when the apoptotic-regulatory proteins are obstructed by pharmacological inhibitors or gene mutations [28]. We demonstrated that xanol decreased the phosphorylation of RIP1, RIP3, and MLKL. An essential biological process in necroptosis is the activation of RIP1, RIP3, and MLKL, which results in necrosome formation, MLKL oligomerization, and membrane translocation [29]. Apoptotic cell death can switch to necroptotic cell death when the caspase-dependent apoptotic pathway is blocked [30]. Our data suggest that xanol exerted its anticancer effects by inducing apoptosis and inhibiting necroptosis in human OSCC cells.

AKT signaling promotes cell proliferation and metastasis while inhibiting cell cycle arrest and apoptosis in human OSCC [31,32]. Patients with OSCC often have altered AKT signaling [33]. It has been reported that signaling pathway inhibitors are potential therapies for human OSCC [20]. In the present study, we demonstrated that xanol suppressed constitutively active PI3K, AKT, mTOR, and p70S6K in human OSCC cells. The AKT signaling pathway has been demonstrated to regulate autophagy. Autophagy is a self-degradation mechanism that maintains homeostasis and plays a crucial role in both cell viability and cell death [34]. Natural compounds have a wide range of applications in the treatment of tumors because they are inexpensive, highly effective, and have low cytotoxicity [35]. The PI3K/AKT/mTOR pathway has an important function in controlling autophagy and achieving antitumor activity [35]. Erianin is a potential anticancer agent that induces apoptosis and autophagy in human OSCC [36]. We demonstrated that xanol promoted autophagic processes and caused a reduction in the number of autophagosomes. An increasing number of studies have shown that autophagy and apoptosis are cooperative in cancer [37,38]. Our data suggest that xanol promotes apoptotic cell death by increasing autophagy via inhibition of the AKT signaling pathway in human OSCC.

Malignant tumorigenesis is associated with metastasis through enhanced cell migration, extracellular matrix breakdown, and the invasion of other tissues [24,39]. In the present study, xanol suppressed the migration, invasion, and anchorage-independent colony formation of human OSCC cells. Consistent with these results, we found that xanol reduced VEGF and MMP13 expression levels. MMPs play crucial roles in tumorigenesis, invasion, and angiogenesis [36]. IL-11 receptor alpha subunit/glycoprotein 130 receptors induce the AKT signaling pathway and subsequently increase MMP-13-mediated migration and metastasis in human OSCC [40]. Propranolol has also been reported to inhibit cell growth and AKT and VEGF levels in human OSCC [41]. Our data suggested that xanol is a potent compound that inhibits malignant tumorigenesis by inhibiting the AKT signaling pathway in human OSCC.

## 5. Conclusions

In conclusion, we demonstrated that xanol exerts anticancer activity by inhibiting the AKT signaling pathway in human OSCC. Although animal experiments are required to demonstrate the anticancer effect of xanol in vivo in future studies, the present data provide new evidence for the biological effects of xanol in suppressing the proliferation, migration, invasion, and tumorigenesis of human OSCC.

## Data Availability

The data generated during the current study are available from the corresponding author on reasonable request.

## Abbreviations

HPLC	high-performance liquid chromatography
LC3A/B	microtubule-associated protein light chain 3A/B
MTT	3-[4,5-dimethylthiazol-2-yl]-2,5-diphenyltetrazolium bromide
OSCC	oral squamous cell carcinoma
xanol	xanthoangelol
